# Regulation of Ribosomal RNA Production by RNA Polymerase I: Does Elongation Come First?

**DOI:** 10.1155/2012/276948

**Published:** 2012-01-12

**Authors:** Benjamin Albert, Jorge Perez-Fernandez, Isabelle Léger-Silvestre, Olivier Gadal

**Affiliations:** ^1^LBME du CNRS, 118 route de Narbonne, 31000 Toulouse, France; ^2^Laboratoire de Biologie Moleculaire Eucaryote, Université de Toulouse, 118 route de Narbonne, 31000 Toulouse, France; ^3^Universität Regensburg, Biochemie-Zentrum Regensburg (BZR), Lehrstuhl Biochemie III, 93053 Regensburg, Germany

## Abstract

Ribosomal RNA (rRNA) production represents the most active transcription in the cell. Synthesis of the large rRNA precursors (35–47S) can be achieved by up to 150 RNA polymerase I (Pol I) enzymes simultaneously transcribing each rRNA gene. In this paper, we present recent advances made in understanding the regulatory mechanisms that control elongation. Built-in Pol I elongation factors, such as Rpa34/Rpa49 in budding yeast and PAF53/CAST in humans, are instrumental to the extremely high rate of rRNA production per gene. rRNA elongation mechanisms are intrinsically linked to chromatin structure and to the higher-order organization of the rRNA genes (rDNA). Factors such as Hmo1 in yeast and UBF1 in humans are key players in rDNA chromatin structure *in vivo*. Finally, elongation factors known to regulate messengers RNA production by RNA polymerase II are also involved in rRNA production and work cooperatively with Rpa49 *in vivo*.

## 1. Introduction

**Figure 1:**
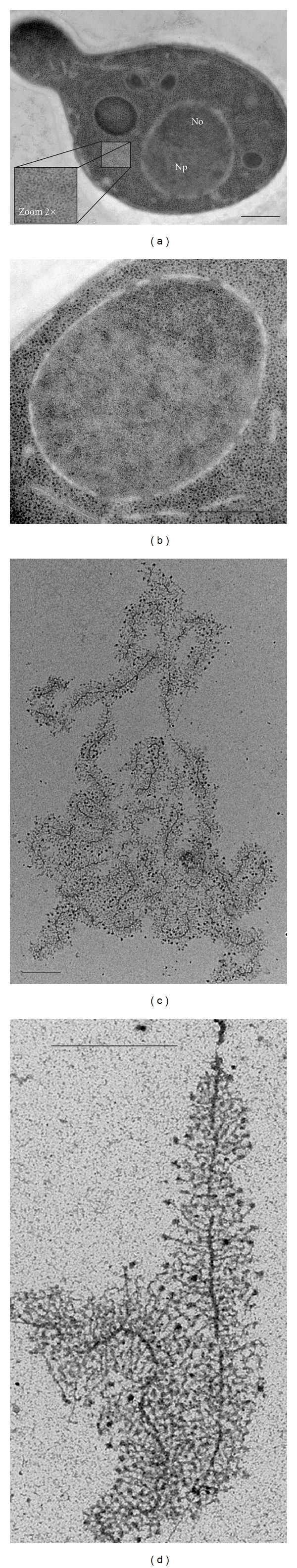
Budding yeast cells and ribosome production. (a) Morphology of *Saccharomyces cerevisiae* cells after cryofixation and freeze substitution. Ribosomes are individually localized in the cytoplasm (see individual ribosomes detected in the zoomed region). In the nucleus, the nucleolus (No) is detected as a large electron-dense region compared with low electron density of the nucleoplasm (Np). (b) Morphology of the nucleolus. The nucleus appears outlined by a double envelope with pores, and the nucleolus is in close contact with the nuclear envelope. In the nucleolus, a dense fibrillar network is visible throughout the nucleolar volume. Granular components are dispersed throughout the rest of the nucleolus. (c) Visualization of active genes in rDNA. Using a mutant strain with a reduced number of rDNA copies (strain NOY1071; 25 rDNA copies), Miller spreading of total nucleolar DNA allowed single-gene analysis of rRNA genes. (d) Quantification of actively transcribed rDNA. Using high magnification, we can detect individual polymerases associated with nascent rRNA. Bars represent 500 nm.

In cell nuclei, three RNA polymerases transcribe the genome. The most importance is placed on RNA polymerase II (Pol II), which is responsible for synthesizing mRNA and a large variety of noncoding RNAs. The vast majority of RNA production in growing cells is carried out by RNA polymerase I (Pol I), which transcribes the precursor of large rRNA, and by RNA polymerase III (Pol III), which transcribes 5S rRNA, tRNA, and some noncoding RNAs. Observation of cryofixed cryosubstituted other sections analyzed by electron microscopy reveals that exponentially growing budding yeast cells contain up to 10^4^ ribosomes per *μ*m^3^ [1], which represents up to 10% of the cytoplasmic volume [2] (Figure 1(a)). 

An early step in ribosome biogenesis is initiated by the extremely high transcriptional activity of Pol I and occurs in the largest nuclear domain, the nucleolus (Figure 1(b)). Electron microscopy of nuclear/nucleolar chromatin dispersed by Miller spreading allowed rRNA gene transcription and cotranscriptional assembly to be visualized directly at the single gene level [3] (Figure 1(d)). rDNA is organized in head-to-tail tandem arrays of rRNA genes [4] contained in budding yeast between 100 and 200 copies per cell [5], and from 200 to 300 per mammalian haploid genome [6]. Analysis of transcribed ribosomal DNA (rDNA) after Miller spreading revealed that up to 150 Pol I enzymes simultaneously transcribe rRNA genes in mutant with only 25 rRNA genes [1, 7] (Figures 1(c) and 1(d)). Importantly, despite being the most highly transcribed genes of the genome, rDNA is subject to epigenetic regulation, and only some rRNA genes are transcriptionally active [8]. In this paper, we will focus on recent advances made in understanding the regulation of Pol I activity, including elongation in the context of ribosome assembly. 

## 2. Is Regulation of Pol I Initiation the Only Regulated Step in rRNA Production *In Vivo*?

**Figure 2:**
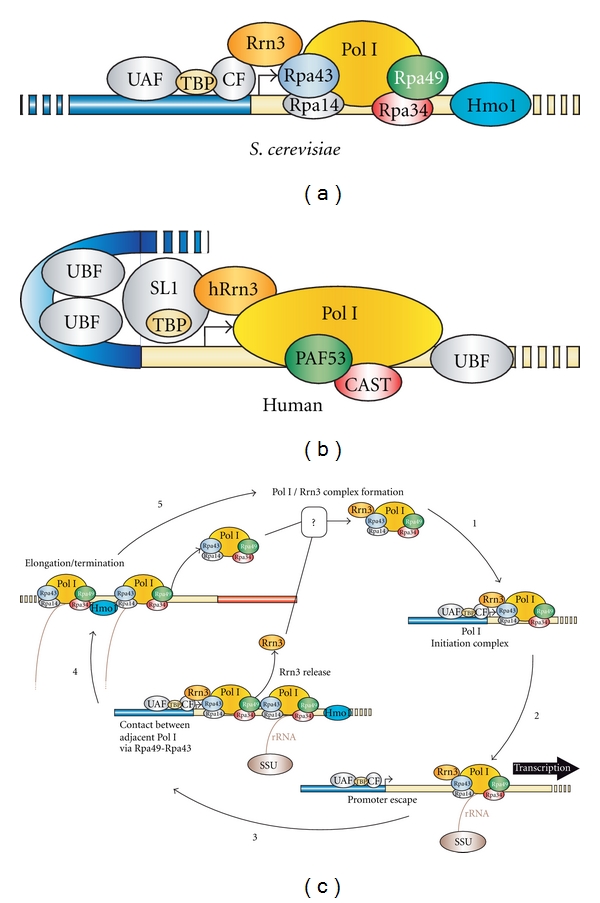
Schematic representation of the Pol I transcription cycle. Simplified composition of the Pol I preinitiation complex (PIC) in (a) budding yeast and (b) human cells. The Pol I transcription cycle in budding yeast. (1) Recruitment of Rrn3/Pol I onto an rDNA promoter associated with the SL1 and UAF complex allows PIC formation. (2) Promoter escape and rRNA synthesis are coupled with cotranscriptional recruitment of the SSU processome. (3) Rrn3 dissociation is achieved by the formation of an adjacent PIC. Pol I subunits Rpa49 and Rpa43 from the adjacent polymerases promote Rrn3 release from the transcribing Pol I. (4) Pol I transcription of rRNA is coupled with nascent rRNA processing and termination. (5) Pol I holoenzyme is recycled by reassociation with Rrn3, an as yet uncharacterized regulatory process. Hmo1 function during elongation remains to be clarified, but is revealed by a tight genetic interaction with Pol I elongation mutant *rpa49*Δ (4 and 5).

Eukaryotic RNA polymerases are able to recognize promoters only when these sequence elements are associated with specific initiation factors. Pol I initiation factors have been characterized for both humans and yeast (Figure 2(a)). In mammals, selectivity factor 1 (SL1) in humans and TIF-1B in mice are composed of the TATA-binding protein (TBP) and four TBP-associated factors (TAFs), bound to the core promoter [9–14]. Upstream binding factor (UBF) acts as a dimer and induces a loop formation called the enhanceosome, which brings the activating sequence into close proximity with the core promoter element [15, 16]. UBF binding stabilizes the association of SL1/TIF-1B with promoter elements [9]. A recent study suggested that UBF bound after SL1 binding and during promoter escape by Pol I [17]. UBF is also bound to the transcribed region [18] and can regulate Pol I elongation [19]. Additionally, UBF and SL1 are regulated by posttranslational modifications. Active Pol I enzymes are associated with numerous other factors such as TFIIH, protein kinase CK2, nuclear actin, nuclear myosin 1 (NM1), chromatin modifiers G9a and SIRT7 and with proteins involved in replication and DNA repair: Ku70/80, proliferating cell nuclear antigen, and CSB. For most factors, mechanistical insights are lacking (see [20] for a recent review).

In budding yeast, a core factor (CF) associates with the Pol I promoter, and this binding is stabilized via TBP by an upstream-associated factor, or UAF [21–27]. CF and SL1 are likely to be functionally equivalent. In contrast, yeast UAF and mammalian UBF both interact with upstream stimulatory elements but have very different functions. UBF1 also regulates Pol I elongation [19]. The *S. cerevisiae* HMG-Box protein, Hmo1, is associated with the Pol I-transcribed region and is able to rescue growth of the Pol I elongation mutant *rpa49*Δ [28]. Therefore, UBF1 and Hmo1 might have a conserved function in stimulating Pol I elongation (Albert et al. submitted).

Surprisingly, both human and yeast Pol I enzymes are unable to initiate productive RNA synthesis with only promoter-bound factors [29, 30]. Only a minor fraction of free Pol I is associated with an additional initiation factor: Rrn3 in yeast, hRrn3 in humans, or TIF-1A in mice (Figure 2(b)). When Pol I associates with one of these factors, it recognizes the promoter-bound factors and forms a preinitiation complex (PIC) [30–35].

The amount of Pol I-Rrn3p complexes represents a limiting step in transcription initiation, but how this association is achieved and regulated remains a major research topic [36]. Numerous signaling pathways target Pol I activity *in vivo*. The target of rapamycin complex 1 (TORC1) regulates ribosome production in response to nutriment availability [37]. Upon inhibition of TORC1 by rapamycin or during stationary phase, the amount of Pol I-Rrn3 complex drops [31, 38, 39]. The regulatory function of Rrn3's association with Pol I was demonstrated by producing an artificial fusion protein of Rrn3 joined to its interacting Pol I subunit, Rpa43. In a partially purified *in vitro* system, this fusion, called CARA, led to a constitutively active Pol I even during stress, showing that Pol I complexed with Rrn3 is initiation competent even under conditions known to inhibit ribosome production [39]. This initial observation suggested that a deregulated initiation event is sufficient to generate constitutively active Pol I *in vivo*. However, other findings are now challenging this initial regulatory model based on the availability of an Rrn3-Pol I complex. Rrn3 function is not restricted to initiation only, and it is also involved in a postinitiation step of the Pol I transcription cycle. Rrn3 is released from Pol I during postinitiation, and this process requires Rpa49, another Pol I-specific subunit [40]. In the absence of Rpa49, the CARA mutant is not viable [40]. Therefore, when Rrn3 is physically tethered to Pol I, Rpa49 function becomes essential [40], which suggests the existence of a functional interaction between Rpa49 and Rrn3 after Pol I recruitment [1]. Initial studies suggested that the interaction between Rrn3 and another Pol I-specific subunit, Rpa43, is regulated by phosphorylation [34, 41]. A mutational analysis of Pol I did not reveal the specific residues involved in this regulation but did not exclude the involvement of phosphorylation [42]. Recent works from the Tschochner's Laboratory have demonstrated that Rrn3 is destabilized by a PEST domain, a peptide sequence rich in proline (P), glutamate (E), serine (S), and threonine (T), in its N-terminal domain [43, 44]. A nondegradable form of Rrn3, missing this PEST motif, attenuated the reduction in initiation competent Pol I-Rrn3p complexes observed upon nutrient depletion. Such a mutation should mimic the CARA mutant phenotype. Unfortunately, this non-degradable form of Rrn3 associated with Pol I has not been tested *in vitro* in a partially purified extract. Nevertheless, in this background, rRNA synthesis was downregulated *in vivo* upon nutrient depletion [44]. Additionally, although levels of the Rrn3-Pol I complex are depleted during stress, the amount remaining is sufficient to observe ongoing initiation events. Therefore, Pol I activity is not regulated only by the initiation competent Rrn3-Pol I complex, but is likely to be influenced by nascent ribosomal assembly. An elegant study suggested that downregulation of ribosomal protein production could also result in a rapid decay of newly made rRNA *in vivo* [45]. Indeed, Sch9, which acts downstream of TORC1, targets ribosomal protein gene (RPG) transcription as well as rRNA production by Pol I [46, 47]. Rrn3 might also impact rRNA processing since a mutant of Cbf5, the pseudouridine synthetase that modifies rRNA, is rescued by Rrn3 overexpression [48]. Along the same lines, accumulation of RPG mRNA in the CARA mutant background was resistant to repression by TORC1 inhibition [39]. In fission yeast, a subunit of the Rrn7 core factor also binds RPG promoters, suggesting a coupling between rRNA production and RPG transcription [49]. In budding yeast, Hmo1, *bona fide* Pol I transcription factors, also bind most RPG promoters [50]. Stochiometric production of all ribosomal constituents is tightly controlled and is probably achieved at multiple levels [2]. *Is Pol I initiation the only regulated step in rRNA production in vivo?* Although the association of Rrn3 with Pol I is a very important regulatory step, it is only one of the numerous pathways that regulate Pol I activity. In this paper, we will extensively describe how the rRNA elongation step might be regulated to integrate all the complex processes necessary to achieve this early step of ribosome assembly.

## 3. Early Ribosome Assembly Occurs during rDNA Transcription

RNA synthesis in the nucleus is invariably coupled with the recruitment of specific proteins shortly after synthesis and leads to the formation of large ribonucleoproteins (RNPs). Strikingly, the fate of the RNA depends on the RNA polymerase synthesizing the transcript. Through their association with the transcribing polymerase appropriate RNA-interacting proteins are driven into the local proximity of the newly synthesized RNA. The COOH-terminal domain (CTD) of the largest subunit of Pol II is the best example of this mechanism [51]. The CTD can recruit the pre-mRNA capping, splicing, and 3′-processing machinery, which are then tethered together with the pre-mRNA. Often, the same factors affecting early RNP assembly, maturation, and export also regulate Pol II elongation, which effectively bridges these processes [52, 53]. Cotranscriptional assemblies of RNP particles are well known to impact the fate of the transcribed RNA. Even more important, polymerase elongation rates can determine the nature of mature mRNA products, as shown by alternative splicing that depends on the elongation rate [54]. 

Pol I transcription provided the first example of cotranscriptional RNP particle assembly [3]. Paradoxically, the intimate connection between early RNP assembly and Pol I elongation has been suggested only recently. From Oskar Miller's original 1969 description of a transcribed rRNA gene as a Christmas tree, in which the nascent RNA cotranscriptionally assembled with maturation factors that appeared as decorating “terminal balls,” 33 years elapsed before the molecular nature of the terminal balls in budding yeast was fully unveiled by the laboratories of Ann Beyer and Susan Baserga [3]. The terminal balls were renamed SSU processomes [55] and are early preribosomal particles, which contain U3 snoRNA and a set of proteins called the UTPs (U three proteins). An intimate relationship between early assembly and transcription was then suggested from a study of a UTP subgroup, the tUTPs, for transcription-UTP [56]. The tUTPs form a complex with a protein composition very similar to the UTP-A complex [57] with only one distinction: the presence of either Utp5 or Pol5, respectively. tUTP/UTP-A is recruited to the chromatin independently of transcription; is required for efficient accumulation of Pol I transcripts; has been suggested to be essential for Pol I transcription in a run-on assay [56]. Alternatively, tUTP was suggested to be required for early pre-rRNA stabilization. In the absence of being complexed with tUTP/UTP-A, nascent RNA transcripts are targeted by TRAMP (Trf4/Air2/Mtr4p Polyadenylation) complex and degraded by the nuclear exosome [58]. In human cells, recruitment of human tUTP orthologs to NORs (*nucleolus organizer regions*) occurs independently of transcription but depends on the protein UBF [59]. Miller spreads have revealed that nascent rRNAs are not only assembled cotranscriptionally with a large set of proteins, but are also cleaved cotranscriptionally in *E. coli* [60], *Dyctiostelium *[61], and budding yeast [62]. This cotranscriptional rRNA cleavage has been independently confirmed and quantified using *in vivo* labeling approaches [63]. Following tUTP/UTP-A recruitment, cotranscriptional assembly is a stepwise and highly hierarchical process [64] that utilizes preexisting autonomous building blocks. These entities sequentially interact with the pre-rRNA and exhibit different interdependencies with respect to each other. This model has been validated and extended by different groups [65].

## 4. Pol I Elongation Rate, rRNA Cotranscriptional Maturation, and Topological Stress

When driven by a strong Pol II promoter, ribosomal DNA can be transcribed by Pol II [66]. Functional ribosomes can ultimately be produced without Pol I, but this is accompanied by a drastic phenotype. Yeast lacking Pol I activity can survive with such an artificial rDNA construct but grow very poorly (i.e., have a doubling time 4 to 5 times longer than normal) and have a massively altered nucleolar and nuclear morphologies [67, 68]. Despite being not strictly essential, the Pol I elongation rate must be properly controlled for efficient ribosome production.

Biochemical purification of Pol I activity copurified a large fraction of the rRNA maturation machinery [69]. Early maturation factors such as Prp43, Nop56, Nop58, or Gno1 have been shown by two-hybrid assay to be physically tethered to the Pol I-specific subunit Rpa34 [40]. The loading of tUTP/UTP-A onto nascent transcripts is facilitated by its association with Pol I promoters prior to pre-rRNA [56, 59] and is required either for transcription or for stabilizing nascent transcripts [58]. The first direct evidence that elongation rate regulation is required for proper ribosomal assembly came from a study of an elongation mutant by David Schneider in the laboratory of Masayasu Nomura [70]. In a mutant bearing a single substitution (*rpa135-D784G*) in the second largest subunit of Pol I, near the active center of the enzyme, rRNA processing was affected with the greatest defect occurring in the 60S assembly pathway [70]. This suggested that the stochiometric production of the 40S and 60S subunits depends on the elongation rate of Pol I.

The structural analysis comparing the three nuclear RNA polymerases revealed one feature that distinguished Pol I from the other two (Pol II and Pol III): the presence of the heterodimer Rpa49/Rpa34 [71]. Rpa49 and Rpa34 form a heterodimer that is structurally similar to the one formed by two Pol II transcription factors, TFIIE and TFIIF. The hPAF53/CAST heterodimer is the human ortholog of Rpa49/Rpa34 [72]. We recently uncovered how Rpa49 releases Rrn3 from Pol I during elongation, despite the diametric opposition of these proteins in the Pol I complex [1, 71]. With such a configuration, Rpa49 and Rrn3 are unlikely to interact directly with each other in the Pol I complex. However, the very high loading rate of Pol I per rRNA gene leads to extensive contact between adjacent Pol I enzymes (Figure 1(d)). Interactions between adjacent Pol I enzymes along the rDNA place Rpa49 and Rpa43 in close proximity, thus allowing Rpa49 to interact with the Rpa43 subunit on a nearby Pol I to promote the release of Rrn3 from Rpa43 [1]. In our model, the Pol I/Rrn3 complex dissociates only after another Pol I starts transcribing rRNA and reflects cooperativity between Pol I enzymes (Figure 2(b)). When we measured distribution of Pol I along rDNA using Miller spreading, we observed a striking degree of enzyme clustering, with 60% of the enzymes in direct contact, which is compatible with our model. Additionally, this clustering was inhibited by deletion of the Rpa49 gene. *What are the consequences of this clustering?* By opening the DNA duplex, DNA in front of RNA polymerase becomes overwound, or positively supercoiled; the DNA behind the polymerase becomes underwound, or negatively supercoiled [73]. Importantly, when multiple polymerases are in close proximity, topological distortion of the DNA duplex in front of and behind each adjacent polymerase would be compensated, resulting in topological stress only in front of the first polymerase, and after the last. Therefore, the absence of clustering of adjacent polymerases in the *rpa49*Δ mutant should lead to massive rDNA supercoiling. Indeed, Rpa49 deletion has a tight genetic relationship with the two type I yeast topoisomerases: Top1 and Top3 [40, 74]. Top3, in complex with Sgs1, is required for the stability of rDNA *in vivo* and is genetically linked to Rpa49 [74, 75]. Top1 is also involved in rDNA stability [76] and seems to be directly involved in rRNA production. Rpa34 has also been found to interact directly with Top1 in two-hybrid assays [40]. 

The extremely high transcription rate of Pol I should lead to extensive torsional stress on the rDNA template.* Is topological stress a selective feature of Pol I transcription?* A good answer to this question came from the study using actinomycin D, a DNA-intercalating agent widely used *in vivo* as a Pol I inhibitor in metazoan cells. The transcription of rRNA genes in mammalian cells is about 50–100-fold more sensitive to actinomycin D than the synthesis of small RNAs and heterogeneous nuclear RNA [77]. However, Pol I-selective inhibition by actinomycin D is not inherent to the Pol I transcriptional machinery. Actinomycin D has no effect at low concentrations *in vitro* or in a transfected reporter system [78]. In fact, low levels of actinomycin D stimulate Pol I rRNA initiation events *in vivo*; Pol I elongation, however, is strongly inhibited [79]. Such surprising activity can be explained by the ability of low actinomycin D concentrations to stabilize DNA/topoisomerase interactions [80]. The specific inhibition of rRNA synthesis is at least in part due to the close relationship between topoisomerase and Pol I activities *in vivo*. 

In yeast, a *top1* deletion mutant led to the formation of R-loops (DNA-RNA hybrids) within transcribed rRNA genes [81]. These rRNA production defects, with accumulation of rRNA intermediates ending in G-rich stretches of the 18S rRNA, are similar to those observed in the Pol I elongation mutants *rpa135-D784G* and* rpa49*Δ (discussed in [70, 81]). Additionally, it was reported that the maximum transcription rate of rDNA by Pol I in a *top1* deletion mutant generates massive negative supercoiling of rDNA template, which was revealed by the presence of DNA template melting in an A-T-rich region of rDNA that was detectable in Miller spreads [82]. Top2 is essential for growth, but *top2* mutants primarily succumb to mitotic failure. Top2 function in rRNA transcription can be studied in Top2 mutants kept in G1 phase and prevented from entering mitosis. Top1 and Top2 cooperate in Pol I transcription [83] but in contrast to a *top1* deletion mutant, a *top2-ts* mutant accumulates positive supercoiling, which inhibits elongation by Pol I [82]. The difference between these Top1 and Top2 phenotypes is surprising since both topoisomerases are able to relax positively and negatively supercoiled DNA. The difference may be related to the intrinsic properties of each enzyme and to the ability of the chromatin to transiently absorb either negative or positive torsional stress [84]. Top1 is a torque-sensitive topoisomerase with a poor ability to relax chromatin. It functions mainly in the relaxation of the negative supercoiling produced in the wake of Pol I [85, 86]. In contrast, Top2 is likely to be more efficient in relaxing the positive supercoiling produced ahead of Pol I, because no sink for torsional stress, such as melting of the DNA template, exists in this case [84]. Finally, genetic interactions between Top1 and Trf4/Trf5, two members of the TRAMP complex [87], have been reported previously, and these might be explained by the presence of unresolved R-loops that accumulate in the absence of nuclear rRNA degradation machinery. 

## 5. Epigenetic Regulation and Chromatin Status of rDNA

In eukaryotes, rRNA genes are either organized in a closed chromatin state in which they are transcriptionally inactive in transcription or are in an open chromatin state [8]. In mammals, inactive rRNA genes are subject to DNA methylation (for review, see [20]). In budding yeast, which lacks DNA methylation, a proportion of rRNA genes is also transcriptionally repressed.

Active rDNA with an open chromatin structure can be distinguished from inactive rRNA genes by psoralen crosslinking. Psoralen is an intercalating reagent which crosslinks to DNA under UV irradiation. The nucleosomes associated with inactive DNA lock the DNA topology and prevent psoralens from crosslinking to DNA. When rDNA is subjected to psoralen treatment and crosslinking, two types of bands are detected: a slow- and a fast-migrating band [8, 88]. The molecular identity of the proteins associated with both types of rDNA molecules was unambiguously established using psoralen combined with ChEC (chromatin endogenous cleavage) [89, 90]. In ChEC methods, rDNA-associated proteins are expressed as recombinant proteins fused to micrococcal nuclease (MNase). After formaldehyde crosslinking and psoralen treatment, MNase is activated and cleaves either the fast- or slow-migrating rDNA band. The fast-migrating band corresponds to transcriptionally inactive rDNA, which is enriched in nucleosomes [90]. The slow-migrating band is transcriptionally competent rDNA, enriched in Pol I, depleted of histones (compared to genomic DNA), and bound by Hmo1, a yeast protein similar to UBF [90]. In budding yeast, the number of rRNA genes in open chromatin seems not to be a major regulatory determinant for Pol I activity [7]. rDNA copy number can change from cell to cell as a result of unequal crossing over and ranges between 100 and 200 repeats in a population [91]. Fob1 is required for this chromosomal instability [92]. In the absence of Fob1, cell populations with a stable number of rDNA repeats could be generated [7]. With 42 copies, or even more so with only 25 copies, most rRNA genes were active and in an open chromatin state. With an rDNA copy number as low as 25 actively transcribed rRNA genes, growth was not affected, but more polymerases were loaded on each active gene [7]. 

During the cell cycle, the ratio of open to closed rDNA changes. Newly replicated rDNA becomes psoralen inaccessible and shows nucleosome assembly on both strands after the passage of the replication forks [93]. Following replication, the amount of open chromatin was found to steadily increase at all stages of the cell cycle, including during cell cycle arrest [94]. This increase required Pol I activity. The maintenance of the open chromatin state did not require Pol I activity, but Hmo1 inhibited replication-independent nucleosome assembly [94]. 

In sharp contrast to ChEC data, chromatin immunoprecipitation (ChIP) analyses targeting H3 and H2B histones in mutant strains harboring most rRNA genes in an open chromatin state (42 or 25 rDNA copies) strongly suggested that significant quantities of histone molecules are present on active rRNA genes [95] (Albert et al. Submitted). 

Both ChIP and psoralen-ChEC have some intrinsic limitations. ChIP is known to be sensitive to background binding, which can lead to false-positive detection. Conversely, ChEC is prone to false-negative detection [89]. After MNase activation, MNase fusion proteins are released from genomic DNA after cleavage of the DNA molecule. These released proteins are then able to cleave genomic DNA at nonspecific sites [89]. ChEC experiments must be performed in a carefully controlled time-course. Due to the abundance of histones molecules, cleavage time is kept bellow 15 minutes [90]. Open rRNA genes appear more resistant to MNase digestion than genes in closed rDNA or naked plasmid DNA, but cleavage is detectable [90]. With such experimental limitations, ChEC combined with psoralen can be used to compare relative levels of histone enrichment, but cannot be used to conclude whether histone is present or absent on open chromatin.

**Figure 3:**
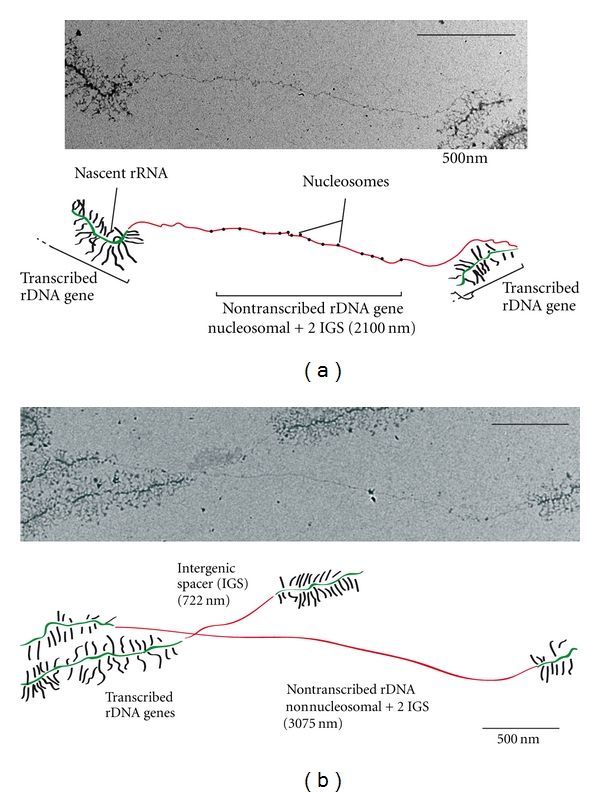
Miller spreading of nontranscribed rDNA. Single-gene analysis of nontranscribed rRNA genes reveals nucleosomal (a) and nonnucleosomal (b) organization. Chromatin spreading (upper panel) and a schematic representation of rDNA spreading (lower panel) are shown from strain NOY1071, bearing 25 rDNA copies. Transcribed rRNA genes (green) are identified by high Pol I density, and nascent rRNAs are shown in black. Non-transcribed regions are depicted in red. Intergenic spaces (IGSs) are short (600 nm) and can be easily distinguished from inactive rRNA genes. Inactive genes are flanked by two IGSs. Due to the DNA wrapping around nucleosomes, nontranscribed genes associated with nucleosomes appear shorter (2,100 nm − (2 × 600 nm) = 900 nm) than genes devoid of nucleosomes (3,100 nm − (2 × 600) = 1,900 nm). With 15 nucleosomes detected and each wrapped around 146 bp, we observed a length reduction of approximately 1,000 nm.

Due to such intrinsic technical limitations, the exact composition of rDNA in the open chromatin state is still widely debated [90, 95]. The analysis of actively transcribed versus untranscribed rDNA can also be performed using Miller spreading (see Figure 1(c)). However, quantifying the ratio of active versus inactive rRNA genes is intrinsically biased, as it underestimates the fraction of inactive rDNA. Transcriptionally inactive rRNA genes are not directly detectable, but can be indirectly visualized because they are flanked by active rRNA genes. This method allows rRNA genes to be characterized at the single-gene level. From such analyses, two important conclusions were reached: nucleosomes are not detectable on actively transcribed genes (data not shown) and nucleosome structures are detectable on some inactive rDNA genes but not all (Figure 3). 

The presence of histones on open chromatin, as detected by ChIP, contrasts sharply with the absence or strong depletion of nucleosomes on open chromatin that is observed with psoralen crosslinking, ChEC, and Miller spreading. These observations are not incompatible if one considers that psoralen crosslinking indicates the presence of canonical nucleosomes, whereas ChIP analysis reveals presence of histone molecules. Histones might still be present on open rDNA copies, but a large body of evidence establishes that they are not arranged as canonical nucleosomes impermeable to psoralen. Alternative nucleosome structures have been described and occur specifically when DNA supercoiling is altered [96]. This observation agrees with older biochemical studies demonstrating that despite the absence of detectable beaded nucleosomes on active rRNA genes, the protein constituents of nucleosomes may still be present [8]. Moreover, by combining reagent accessibility analyses and electron microscopy of rDNA from *Physarum polycephalum*, the existence on active rRNA genes of an alternative nucleosome structure called the lexosome was suggested some time ago [97, 98]. The lexosome is an altered nucleosome specifically located on actively transcribed regions, which has properties that facilitate transcription. In a lexosome configuration, the histone-DNA interactions are different than those in intact nucleosomes and allow psoralen to access DNA. Therefore, even if this alternative structure was not confirmed when tested for *in vitro* transcription [99], the lexosome represents an attractive model that is consistent with the ChIP data, the psoralen-crosslinking results, and the electron microscopy images produced in our studies as well as those of other research teams. Similarly, the altered topology of actively transcribed rDNA might lead to other alternative histone configurations [96].

## 6. Regulator of Pol I Elongation

To date, most factors known to regulate Pol I elongation were characterized previously as Pol II elongation factors. Such dual functions make interpretation of phenotype difficult, since an indirect effect via Pol II is difficult to exclude. However, some Pol I elongation factors are now well characterized.

Spt5 copurifies with Pol I and has been shown to be associated with rDNA *in vivo* [100]. Spt5 is an evolutionarily conserved elongation factor with homologs found in eubacteria (NusG) and in *archaea* (RpoE) [101, 102]. Multisubunit RNA polymerases have the ability to stably interact with DNA through a structural feature called the DNA clamp. Prior to interacting with DNA, the DNA clamp must be in an open configuration and closed for processive elongation. Spt5/NusG can close the DNA clamp, making the polymerase able to carry out processive elongation [103]. Spt5 interacts with multiple Pol I subunits and Rrn3, and an Spt5 mutant (*spt5-C292R*) suppresses the growth defect of the *rpa49*Δ Pol I mutant [104]. Phenotypic analysis of rDNA transcription in the Spt5 mutant suggested that it positively and negatively regulates Pol I functions [105]. Other factors that interact with Spt5, such as Spt4, Paf1 and Spt6, have also been suggested to regulate Pol I. An *spt4* deletion mutant led to decreased rDNA copy numbers, and an rRNA processing defect [100]. The Paf1, complex interacts physically with Spt5 [106] and is involved in stimulating Pol I elongation [107]. Spt6 is a histone chaperone that might also be a good candidate to regulate Pol I *in vivo*. In addition, the FACT complex stimulates Pol I activity in human cells [108]. FACT consists of Spt16 and Pob3, interacts with Spt5 in yeast [106], and is genetically linked to *rpa49*Δ [109]. Another Pol II transcription factor has been proposed to regulate Pol I: the Elongator, a six-subunit complex, conserved between yeast and mammals. In African trypanosomes, mutation or down-regulation of the Elp3b subunit of the complex results in increased synthesis of rRNA by Pol I [110]. 

The growing list of Pol II elongation factors that also regulate Pol I activity is interesting, but some mechanistic insights are still lacking. The elongation mechanisms of Pol II and Pol I are very different. Pol II carries regulators via interactions with CTD [111]. With few Pol II enzymes acting simultaneously on transcribed genes, the stoichiometric association of elongation factors with Pol II results in a density of about one elongation factor per gene. In contrast, a large number of Pol I enzymes can transcribe a single rRNA gene. Thus, it is difficult to imagine that Pol II elongation factors would be stochiometrically bound to each elongating Pol I. Therefore, it seems unlikely that Pol II and Pol I complexes use similar mechanisms of action or factor recruitment strategies. It remains to be understood how the same elongation factors can act in two very different transcription systems.

One factor can now be defined as a *bona fide* Pol I elongation factor, the nucleolin, called Nsr1 in budding yeast and Gar2 in fission yeast. Following nucleolin's first identification as an abundant nucleolar protein, it has been implicated in numerous cellular processes [112, 113]. Among them, it is clearly involved in ribosome biogenesis. Nucleolin is required for early rRNA-processing events [114] and for Pol I activity through a nucleosomal template [115]. Nucleolin has a histone chaperone activity and stimulates transcription by a mechanism reminiscent of the activity of the FACT complex [116]. Nucleolin is clearly an important factor to understand the interplay between rDNA chromatin, Pol I transcription, and cotranscriptional rRNA processing.

## 7. Concluding Remarks

In this paper, we tried to focus on the unanswered questions of rRNA production, rather than make an exhaustive review of the large body of work addressing regulation of this complex multistep process. We still know little about how cells adjust the production of each ribosomal constituent in time and space to allow cotranscriptional assembly of preribosomal particles. The answer to this question probably lies in the existence of highly redundant pathways, which are all designed to coregulate rRNA production by Pol I, ribosomal protein production, and the availability of the ~200 transacting factors required to assemble eukaryotic ribosomes. The discovery of redundant pathways has clearly resulted from the extensive study of budding yeast. Most regulatory pathways affecting rRNA production are not essential for cell growth. Out of 14 Pol I subunits, four are not required for cell growth. However, when double inactivation is performed, their functions can be revealed and studied [74]. We have no doubt that important progress remains to be made in understanding how Pol I is regulated. Because most of the complex interplay between rRNA production, assembly, cleavage, and folding occurs during elongation, we expect that most progress remaining to be made will uncover how rRNA elongation is coupled to rRNA assembly [81]. The central question of the exact structure and composition of open rDNA chromatin remains a major challenge. Pol I elongation is likely to be the most important step in controlling how nascent rRNA is folded and cleaved to yield pre-60S and pre-40S rRNA as they are being assembled into large preribosomes. We propose that elongation is the most regulatable step in rRNA production, making elongation the first target to regulate rRNA production.
